# Primary hyperoxaluria type 1 diagnosis in adult dialysis patients: prediction model assessment in a group of Italian patients

**DOI:** 10.1007/s40620-025-02243-3

**Published:** 2025-06-04

**Authors:** Pietro Manuel Ferraro, Eloisa Arbustini, Diego Bellino, Chiara Caletti, Irene Capelli, Giovanna Capolongo, Maria Rosa Caruso, Paola Cianfrone, Maria Michela D’Alessandro, Marina Di Luca, Giovanni Gambaro, Alessandra Palmisano, Andrea Ranghino, Gaia Santagostino Barbone, Francesca Viazzi, Loretta Zambianchi, Giorgia Mandrile

**Affiliations:** 1 Section of Nephrology, Department of Medicine, Università degli Studi di Verona, Piazzale L.A. Scuro 10, 37134 Verona, Italy; 2 Center for Inherited Cardiovascular Diseases, Scientific Department, IRCCS Foundation, Policlinico San Matteo, Pavia, Italy; 3 Clinica Nefrologica Dialisi e Trapianto, IRCCS Ospedale Policlinico San Martino, Genoa, Italy; 4 Nephrology, Dialysis and Renal Transplant Unit, IRCCS Azienda Ospedaliero-Universitaria di Bologna, Alma Mater Studiorum, University of Bologna, Bologna, Italy; 5 Department of Translational Medical Sciences, Unit of Nephrology, University of Campania “Luigi Vanvitelli”, Naples, Italy; 6 Papa Giovanni XXIII Hospital, Bergamo, Italy; 7 Nephrology and Dialysis Unit, Magna Graecia University Hospital, Catanzaro, Italy; 8 Pediatria ad Indirizzo Nefrologico e Dialisi, P.O. G. Di Cristina, A.R.N.A.S. Ospedali Civico Di Cristina Benfratelli, Palermo, Italy; 9 Department of Nephrology and Dialysis, Ospedale Santa Croce, Azienda Sanitaria Territoriale n 1, Fano, Pesaro-Urbino Italy; 10 Nephrology Unit, Department of Medicine and Surgery, University Hospital of Parma, Parma, Italy; 11 Nephrology, Dialysis and Renal Transplantation Unit, Azienda Ospedaliero Universitaria delle Marche, Ancona, Italy; 12 Department of Clinical, Special and Dental Sciences, Marche Polytechnic University, Ancona, Italy; 13 Nefrologia e Dialisi, Ospedale Bassini, ASST Nord Milano, Cinisello Balsamo, Milan, Italy; 14 Section of Nephrology, Department of Medicine, Ospedale Nuovo Morgagni, Forlì, Italy; 15 Genetic Unit and Thalassemia Center, San Luigi Gonzaga University Hospital, Orbassano, Italy

**Keywords:** Primary hyperoxaluria, PH1, Diagnostic algorithm, Red flags

## Abstract

**Background:**

To increase the diagnostic rate of primary hyperoxaluria type 1 (PH1) in the adult dialysis setting, a prediction model based on five readily available clinical parameters was recently developed and validated in an adult hemodialysis population. To further test the prediction model in clinical practice, this case series describes the retrospective application of the diagnostic algorithm in a group of adult dialysis patients with PH1 treated at different Italian nephrology centers.

**Methods:**

Between January and May 2023, adult patients (≥ 18 years) undergoing chronic hemodialysis with a genetic diagnosis of PH1, followed at 14 Italian nephrology centers, were selected for the retrospective application of the prediction model.

**Results:**

The presence of at least one red flag of the diagnostic algorithm was reported in most patients (14 out of 15; 93%), two red flags were present in four patients (27%), and three red flags in two patients (13%). A history of active nephrolithiasis was the most common clinical feature (87% of patients), followed by early dialysis initiation, nephrocalcinosis and a family history of CKD (20–27%).

**Conclusions:**

Our study provides further evidence on the real-world application of a simple algorithm, implemented by easily accessible clinical parameters, to be used as a screening tool for diagnosing PH1 in adult patients undergoing dialysis. The successful implementation of this prediction model has the potential to facilitate timely diagnosis, improve patient outcomes, and inform targeted therapeutic interventions in this patient setting.

**Graphical abstract:**

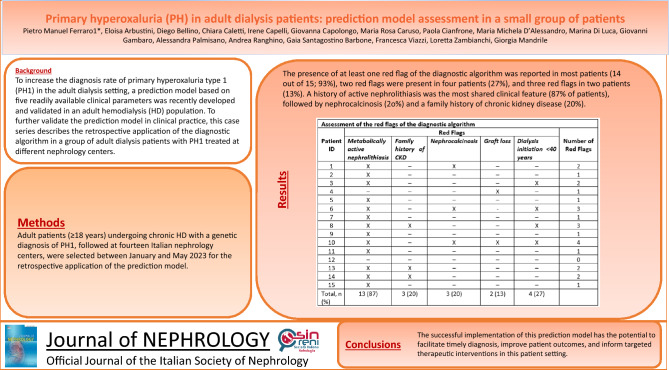

**Supplementary Information:**

The online version contains supplementary material available at 10.1007/s40620-025-02243-3.

## Introduction

Primary hyperoxalurias (PHs) are ultra-rare autosomal recessive inborn errors (prevalence 1–3 per 1,000,000) of hepatic glyoxylate metabolism, characterized by overproduction and elevated urinary excretion of oxalate [1, 2]. Three genetic forms of PH (PH1, PH2 and PH3) have been described, caused by mutations of the *AGXT*, *GRHPR* and *HOGA1* genes, respectively [3, 4]. PH1 is the most frequent and severe PH form, accounting for about 80% of genetically characterized patients [2, 5].

In PH1, the kidneys represent the main target of oxalate crystal accumulation, leading to end-stage kidney disease [6]. As kidney function declines, plasma oxalate levels increase, and systemic oxalosis may develop, causing nephrocalcinosis and kidney failure, as well as osteoskeletal, cardiovascular, ocular, cutaneous, and neurological symptoms [7].

Genetic testing is the gold standard for diagnosing PH, and genetic confirmation and typing of PH are pivotal to managing these patients [8]. Although the onset of PH1 typically occurs in childhood, symptoms can appear later in life and even in adulthood [9, 10]. In adult patients, PH1 presents with phenotypic heterogeneity, ranging from nephrocalcinosis with kidney failure to only occasional stone formation similar to idiopathic stone disease [10]. These factors, along with the rarity of this disorder, are responsible for its underdiagnosis in adult patients, particularly among those undergoing dialysis [4, 11]. In this context, up to 50% of patients have chronic kidney disease (CKD) or kidney failure at diagnosis, and approximately 10% are diagnosed after disease recurrence on kidney allograft [1, 11]. This evidence suggests the fundamental importance of a prompt diagnosis of PH1 to improve patient care and subsequent therapeutic strategies, particularly in the adult setting; it also highlights that the definition and optimal deployment of screening tools for this rare condition represents an unmet need [10].

With the aim of increasing the diagnosis rate of PH1 in the adult dialysis setting, we recently developed and validated a prediction model based on readily available clinical parameters in an adult hemodialysis (HD) population [12]. To test our prediction model in clinical practice, we applied to this case series the diagnostic algorithm developed in a group of adult dialysis patients with PH1 treated in several Italian nephrology centers.

## Patients and methods

Adult patients (≥ 18 years) undergoing chronic HD with a genetic diagnosis of PH1 were selected between January and May 2023 for the retrospective application of our prediction model. Patients were followed in 14 Italian nephrology centers, namely: San Luigi Orbassano Hospital (Turin), Azienda Ospedaliera Ospedali Riuniti Marche Nord (Pesaro), Sant’Orsola Polyclinic (Bologna), San Martino Polyclinic (Genova), ASST Papa Giovanni XXIII (Bergamo), San Matteo Polyclinic (Pavia), Parma hospital (Parma), ASST Nord Milano Bassini Hospital (Milan), G.B. Morgagni L Pierantoni Hospital (Forlì), University Hospital Mater Domini (Catanzaro), University Hospital Paolo Giaccone (Palermo), University Hospital “Luigi Vanvitelli” (Naples), Azienda Ospedaliero Universitaria delle Marche (Ancona), University Hospital of Verona. Baseline demographic characteristics and data from clinical records were collected using the REDCap electronic data capture tool [13]. Patients included in this cohort were not included in our previous studies. Patients with an established alternative diagnosis were excluded.

The patient data review was conducted in accordance with the ethical standards as laid down in the 1964 Declaration of Helsinki and its later amendments and was notified to the Ethics Committee of San Luigi Orbassano Hospital. All participants provided consent to the use of their medical records for research purposes.

### Diagnostic algorithm

As previously described, the prediction model is based on easy-to-collect clinical parameters [Ferraro 2024]. Briefly, it considers five pre-specified “red flags”: (1) active nephrolithiasis (defined as recurrent and/or early onset and/or bilateral and/or with significant family history; based on the patient’s clinical history and including the period before initiation of kidney replacement therapy); (2) presence of nephrocalcinosis; (3) previous graft loss with no established cause; (4) onset of HD before 40 years of age; and (5) family history of CKD (linked to either a sibling or a cousin) [12]. In the validation phase, the algorithm was applied to a cohort of adult patients with PH1 undergoing HD and compared with the general adult HD population, assuming none of these patients were affected by PH1 (in some patients, genetic testing was also performed for further exclusion). The discrimination of the model was high (area under the curve: 0.93; 95% CI 0.86–1.00), and the calibration was appropriate [12]. This suggests that this prediction model can be considered a suitable screening tool to identify adult HD patients with a high likelihood of PH1, who could then undergo further testing to achieve a diagnosis of certainty. The authors recommended a low threshold for action (e.g., obtaining further information in patients with a PH score of at least (1), especially for those patients being evaluated for a kidney transplant [12]. In the present study, we retrospectively applied the prediction model to assess the presence of the five pre-specified “red flags” in our cohort of adult patients with a genetic diagnosis of PH1 undergoing chronic HD. Other suspicion criteria and clinical characteristics were also collected.

## Results

### Patient description

In our cohort of 15 patients, the mean age at PH1 diagnosis was 45 years (Table 1), and the mean age at the last follow-up was 51 years. The most common *AGXT* pathogenic variant was p.Gly170 Arg (10, 66%). More than half of patients (*n* = 9; 60%) were recently diagnosed (< 2 years). Three patients received the diagnosis of PH1 after disease recurrence on kidney allograft (patient ID: 3, 4, 10). To date, one patient (ID: 12) has died from liver cirrhosis. Patient characteristics at diagnosis and last follow-up are summarized in Table 1. Available urinary and plasma oxalate values at baseline and last follow-up are reported in Supplementary Table 1.

**Table 1:** Patient characteristics at diagnosis and last follow-up

Patient ID	Sex	Age at diagnosis (years)	Ethnic origin	Pathogenic variant	Dialysis modality at diagnosis	Time from diagnosis (years)	Dialysis modality at last follow-up
1	Male	33	Pakistani	Homozygote c.33 dupC, p.(Lys12fs)	HD	1	Transplant
2	Male	50	Tunisian	Homozygote c.731 T > C, p.(Ile224 Thr)	HD	1	HD
3	Male	29	Italian	Homozygote c.508G > A, p.(Gly170 Arg)	Transplant	11	Transplant
4	Male	72	Italian	Homozygote c.508G > A, p.(Gly170 Arg)	Transplant	< 1	HD
5	Male	51	Tunisian	Homozygote c.731 T > C, p.(lle244 Thr)	HD	1	HD
6	Male	35	Italian	Homozygote c.322 T > C, p.(Trp108 Arg)	HD	1	HD
7	Male	70	Italian	Homozygote c.508G > A, p.(Gly170 Arg)	PD	1	PD
8	Male	38	Lebanese	Homozygote c.364 C > T, p.(Arg122*)	HD	2	HD
9	Male	31	Albanian	Homozygote c.508G > A, p.(Gly170 Arg)	No KRT	16	No KRT
10	Female	29	Italian	Homozygote c.508G > A, p.(Gly170 Arg)	Transplant	11	Transplant
11	Male	24	Albanian	c.508G > A, p.(Gly170 Arg)/c.242 C > T, p.(Ser81Leu)	No KRT	21	PD
12	Male	62	Italian	Homozygote c.508G > A, p.(Gly170 Arg)	HD	14	HD
13	Male	49	Italian	c.508G > A, p.(Gly170 Arg)/c.33 dupC, p.(Lys12fs)	HD	11	HD
14	Female	52	Italian	Homozygote c.508G > A, p.(Gly170 Arg)	HD	1	HD
15	Female	49	Italian	c.466G > A, p.Gly156 Arg/c.508G > A, p.(Gly170 Arg)	HD	< 1	HD

*HD* hemodialysis; *PD* peritoneal dialysis; *KRT* kidney replacement therapy

### Retrospective application of the prediction model

The majority of patients (*n* = 13; 87%) in our cohort had a history of active nephrolithiasis; nephrocalcinosis and early dialysis initiation were reported in three (20%) and four (27%) patients, respectively (Table 2). Family history of CKD was reported by three patients (20%; none of the patients were consanguineous), while two patients (13%) experienced graft loss (Table 2). Three out of five red flags were reported in two (13%) patients, while two red flags were present in four patients (27%). For one patient, no red flags were reported. Other suspicious criteria were reported for patient 4 (acute kidney injury after kidney transplant, with histological evidence of renal intratubular calcium oxalate deposits), patient 7 (reduced calcium content throughout the segment at skeletal X-ray with osteopetrosis), and patient 12 (acute kidney injury with calcium oxalate crystals found on the kidney biopsy).

**Table 2:** Assessment of the red flags of the diagnostic algorithm

Patient ID	Red flags					Number of red flags
	Metabolically active nephrolithiasis	Family history of CKD	Nephrocalcinosis	Graft loss	Dialysis initiation < 40 years	
1	X	–	X	–	–	2
2	X	–	–	–	–	1
3	X	–	–	–	X	2
4	–	–	–	X	–	1
5	X	–	–	–	–	1
6	X	–	X	-	X	3
7	X	–	–	–	–	1
8	X	X	–	–	X	3
9	X	–	–	–	–	1
10	X	–	X	X	X	4
11	X	–	–	–	–	1
12	–	–	–	–	–	0
13	X	X	–	–	–	2
14	X	X	–	–	–	2
15	X	–	–	–	–	1
Total, *n* (%)	13 (87)	3 (20)	3 (20)	2 (13)	4 (27)	

*CKD* chronic kidney disease

## Discussion

The presentation of primary hyperoxaluria type 1 in adults is more varied in terms of symptoms, timing, and severity than in children [10]. Consequently, a major focus in this setting concerns improving the diagnostic work-up. Indeed, prompt diagnosis of PH1 is essential to prevent downstream complications and optimize therapeutic strategies, considering the presence of (partially) pyridoxine-sensitive mutations, particularly in light of the recent availability of new therapies based on RNA interference [14–17].

A prediction model based on five easy-to-collect clinical parameters (red flags) was recently developed to provide a screening tool to identify adult HD patients with a high likelihood of PH1 [12]. In this study, we retrospectively applied this model to our cohort of 15 adult patients with PH1. We found the presence of at least one red flag in the majority of patients (14 out of 15; 93%), two red flags were present in four patients (27%), and three red flags in two patients (13%). A history of active nephrolithiasis was the most commonly shared clinical feature (87% of patients), followed by early dialysis initiation, nephrocalcinosis and a family history of CKD (20–27%). One patient only in our cohort had no red flags. This is consistent with our previous work, in which, although the diagnostic score effectively discriminated patients with PH1, we still observed some cases with a score of 0 in both the training and validation sets [12]. This could be due to imprecise ascertainment of the clinical red flags or to some events, such as stone formation, going unnoticed.

It is worth mentioning that in North African and Middle Eastern populations, encompassing countries with high consanguinity rates, the prevalence of PH1 is higher than in Europe, the USA and Japan [18]. These represent an “enriched population”, where the disease is most prevalent. According to this evidence, 40% of our patients were of an ethnic origin for which a higher prevalence of the disease has been observed, suggesting that careful attention must be paid to the presence of red flags in patients belonging to the"enriched population".

Altogether, our findings reinforce the idea that our proposed tool is a valuable screening aid, but clinical suspicion is still key to reaching a diagnosis. As previously reported, since this model is intended to be an inexpensive screening tool, it is recommended to request further information in patients with a score ≥ 1, especially in patients evaluated for a kidney transplant [12]. Indeed, given that PH1 is a rare disease, prioritizing sensitivity over specificity is crucial to minimizing missed diagnoses.

## Conclusions

Our study provides further evidence on the real-world application of a simple algorithm built on easily accessible clinical parameters, highlighting that it can be used as a screening tool for PH1 diagnosis in adult patients undergoing dialysis. The implementation of this prediction model may facilitate diagnosis, improve patient management and outcomes, and inform targeted therapeutic interventions. Our findings need further validation in larger cohorts.

## Supplementary Information

Below is the link to the electronic supplementary material.

Supplementary file1 (DOCX 20 KB)

## Data Availability

All data generated or analyzed in this article are included in this article. Further inquiries can be directed at the corresponding author.
