# Supplementary material for: Primary hyperoxaluria type 1 diagnosis in adult dialysis patients: prediction model assessment in a group of Italian patients
Source: J Nephrol. 2025 Jun 4;38(8):2199–204. doi: 10.1007/s40620-025-02243-3 (PMC12630283; doi:10.1007/s40620-025-02243-3)
Supplement: Supplementary file 1 — Supplementary file1 (DOCX 20 KB) [file 40620_2025_2243_MOESM1_ESM.docx]

| **Supplementary Table 1. Urine* and plasma** oxalate values at baseline and last follow-up.** | | | | |
| --- | --- | --- | --- | --- |
| **Patient ID** | **Baseline** | | **Last follow-up** | |
|  | ***Urine oxalate (mg/day)*** | ***Plasma oxalate (mg/L)*** | ***Urine oxalate (mg/day)*** | ***Plasma oxalate (mg/L)*** |
| 1 | 181 | 191 | NA | 58 |
| 2 | NA | 72 | NA | 80 |
| 3 | 75 | 161 | 52 | 45 |
| 4 | 114 | 30 | NA | NA |
| 5 | NA | 175 | NA | NA |
| 6 | NA | 227 | NA | NA |
| 7 | 23 | 54 | NA | 65 |
| 8 | NA | 130 | NA | 78 |
| 9 | 196 | NA | NA | NA |
| 10 | NA | 109 | 23 | NA |
| 11 | NA | ND | NA | 88 |
| 12 | NA | 147 | NA | NA |
| 13 | 93 | 160 | NA | 160 |
| 14 | 181 | 191 | NA | 58 |
| 15 | NA | 126 | NA | 126 |
| 16 | 5 | 219 | NA | 91 |
| NA: not available. * Urine oxalate normal values: <50 mg/day; ** Plasma oxalate normal values: <0.54 mg/L. | | | | |
